# Percutaneous Tibial Nerve Stimulation for Neurogenic Bladder Due to Severe Lumbosacral Disc Herniation

**DOI:** 10.3390/jcm14072262

**Published:** 2025-03-26

**Authors:** Do-Young Kim, Ji-Sung Yeom, Ye-Rim Yun, Joon-Seok Lee, Won-Jeong Ha, In-Hyuk Ha, Yoon Jae Lee, Doori Kim

**Affiliations:** 1 Jaseng Korean Medicine Hospital, Seoul 06110, Republic of Korea; 95kent@jaseng.co.kr (D.-Y.K.);; 2 Department of Public Health Science, Seoul National University, Seoul 08826, Republic of Korea; 3 Jaseng Spine and Joint Research Institute, Jaseng Medical Foundation, 2F Vision Tower, 536 Gangnam-daero, Gangnam-gu, Seoul 06110, Republic of Korea

**Keywords:** case report, herniated disc, neurogenic bladder, PTNS

## Abstract

**Background:** Neurogenic bladder (NB), resulting from neurological disorders, significantly affects quality of life and increases healthcare costs. Although percutaneous tibial nerve stimulation (PTNS) is an established therapy for central nervous system-related lower urinary tract dysfunction (LUTD), its efficacy in treating intervertebral discogenic LUTD remains unexplored. This study presents the first documented case of PTNS applied to NB secondary to severe lumbosacral herniated intervertebral disc (HIVD). **Methods:** A 39-year-old female, hospitalized twice for worsening HIVD, presented with LUTD, including urgency, weak stream, and nocturia. Magnetic resonance imaging confirmed progressive L5-S1 disc extrusion with sacral nerve compression. PTNS, delivered via electronic stimulation through acupuncture needles at SP6 and KI3, was administered daily for 10 days during hospitalization. Symptom scores relating to LUTD, pain, and physical disability were evaluated. **Result:** The American Urological Association symptom score showed significant improvement (from 20 to 6 and 22 to 15 at 12 weeks after the first and second hospitalizations, respectively). Recovery of voiding function was slower during the second hospitalization, possibly due to increased sacral nerve compression and chronic pathologic condition. Pain and functional disability, assessed using the NRS and ODI, improved by approximately 50% (from 55 to 25 and 80 to 45 during the first and second hospitalizations, respectively) and two-thirds (from 66 to 42 and 93 to 66, respectively). **Conclusions:** This case suggests that PTNS may be a viable conservative therapy for HIVD-associated LUTD. Further research is required to elucidate its mechanistic effects and clinical efficacy in peripheral nerve-related bladder dysfunction.

## 1. Introduction

Neurogenic bladder (NB) is a dysfunction of the urinary bladder resulting from neurological disorders affecting the central and/or peripheral nervous system (CNS and PNS, respectively) involved in micturition control [1]. Symptoms vary depending on the specific nerve impairments associated with disorders such as Parkinson’s disease (PD), multiple sclerosis (MS), and spinal cord injury (SCI) [2]. Bladder dysfunction is estimated to affect more than 70% of individuals with PD, MS, or SCI [3,4,5]. Up to 40% of patients with lumbar disc disease exhibit abnormal urodynamic findings, with an even greater proportion reporting voiding symptoms [6]. Individuals with NB experience significantly impaired quality of life, especially due to urgency and incontinence, which contribute to psychological and social issues [7]. The annual healthcare cost of supportive care for NB is estimated at approximately USD 12,219 per patient [8].

Evidence on the management of NB caused by a herniated intervertebral disc (HIVD) remains limited. According to the 2021 American Urological Association (AUA) guidelines, oral medication and percutaneous tibial nerve stimulation (PTNS) are recommended for NB patients presenting with lower urinary tract dysfunction (LUTD), including urgency, frequency, and incontinence [9]. PTNS, a needle-based electrical stimulation technique, modulates bladder function by delivering retrograde neuromodulation to the sacral nerve plexus via the tibial nerve, and is usually employed to treat overactive bladder syndrome [10]. This approach alleviates LUTD with high patient satisfaction and a favourable safety profile [11]. However, in the case of discogenic LUTD, therapeutic application of PTNS for LUTD associated with HIVD-induced nerve compression has not been previously reported.

This study aims to (1) report a case evaluating the effect of PTNS on LUTD associated with HIVD-induced NB and (2) explore the potential relationship between PTNS efficacy and the severity and characteristics of HIVD. This case report presents the first documented treatment of NB symptoms in a patient with severe lumbosacral HIVD using PTNS. This case provides foundational data for future clinical applications in managing bladder dysfunction caused by disc pathology and serves as a basis for large-scale clinical trials and mechanistic studies of PTNS.

## 2. Case Presentation

### 2.1. Patient Characteristics

**Figure 1:**
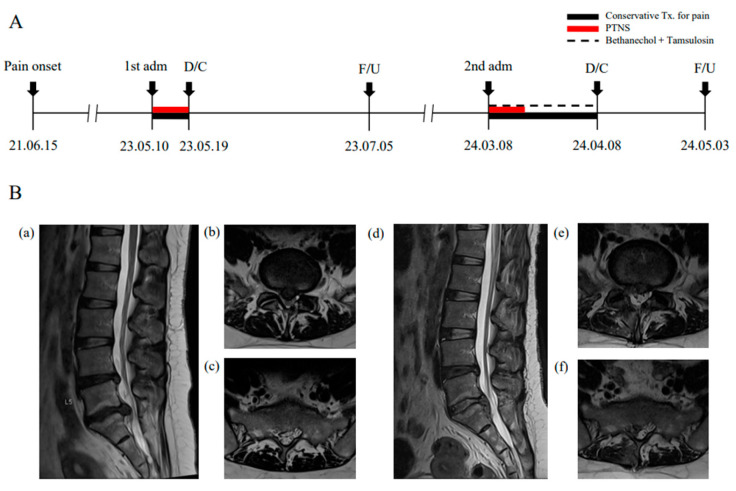
Timeline of the patient’s treatment history with radiological findings. (**A**) Timeline of medical events from pain onset to two hospital admissions, including hospitalization periods, follow-ups, and treatment contents. (**B**) Lumbar spine magnetic resonance imaging (MRI). MRI scans were performed twice on the admission days ((**a**–**c**): first Adm; (**d**–**f**): second Adm). (**a**,**d**) show sagittal T2-weighted images, while (**b**,**e**) and (**c**,**f**) present axial views at the mid-L5 and mid-S1 levels, respectively. Adm—admission; D/C—discharge; F/U—follow-up; PTNS—percutaneous tibial nerve stimulation; Tx.—treatment.

This study reports the treatment and prognosis of a patient who was admitted twice consecutively under the same clinical conditions (Figure 1A). A 39-year-old female (height: 167.3 cm, weight: 72.2 kg) was admitted to the Jaseng Hospital of Korean Medicine on 10 May 2023, with worsening low back pain accompanied by pain and numbness in the right leg, which had initially begun two years prior. On admission, her straight leg raise was 30°/30° with sensory loss in the posterior right leg extending to the sole, lateral foot, and little toe, without muscle weakness in the lower extremities. She also experienced LUTD, including an incomplete voiding sensation, weak urinary stream, urgency, and nocturia, which correlated with pain severity. During her second admission on March 08, 2024, her symptoms were similar, but their intensity had worsened. The patient had not received treatment from other institutions for symptom management and had intermittently taken analgesics for severe pain, as needed. She reported a sedentary lifestyle with no history of smoking or alcohol consumption, and no significant comorbidities or family history of underlying diseases.

### 2.2. Diagnosis and Treatment

The diagnosis of HIVD was confirmed by lumbar spine magnetic resonance imaging, performed twice on the first day of hospitalization (Figure 1A). Initially, HIVD at the L4-5 level (right central disc protrusion) and L5-S1 level (right central disc extrusion) were identified. A second imaging scan revealed inferior migration of the L5-S1 disc with right S1 nerve root compression and spinal canal stenosis (Figure 1B). NB secondary to HIVD was diagnosed based on urinalysis findings and the patient’s medical history, which aligned LUTD symptoms with discogenic pain while ruling out other potential causes. Routine laboratory tests, including blood and urine analyses, showed no significant abnormalities.

PTNS was performed in accordance with the protocol described in a previous study [12], except for small differences in the needle size, puncture points, and treatment frequency. In this study, 0.30 × 30 mm needles (30-gauge, DongBang Co., Seoul, Republic of Korea) were inserted at the right SP6 and KI3 acupoints, selected based on the direction of the HIVD and the tibial nerve distribution. Electrical stimulation was applied at a patient-tolerable intensity of up to 20 Hz for 20 min, an intensity known to be effective in CNS rehabilitation [13]. Correct needle placement was confirmed by observing the large toe flexion. PTNS was administered once daily for 10 consecutive days during both hospital admissions. During the second admission, oral medications (bethanechol [75 mg] and tamsulosin [0.2 mg] daily for a month) were also prescribed due to the severity of symptoms.

For discogenic pain, routine conservative treatments, including acupuncture targeting the lower back and herbal medicine, were administered twice daily (Table 1). The treatment protocols were identical except for the hospitalization length.

### 2.3. Symptom Course

The severity of LUTD was assessed using the AUA urinary symptom score, a 7-item scale (total 0–35 points; higher scores indicate greater severity; 0 to 7 [mild], 8 to 19 [moderate], 20 to 35 [severe]) evaluating voiding dysfunction (incomplete emptying, intermittency, weak stream, and straining) and storage (frequency, urgency, and nocturia) [14]. For discogenic pain, the numeric rating scale (NRS) and Oswestry Disability Index (ODI) were used to evaluate pain severity and physical function, respectively. All measurements were assessed at 10 days, 8 weeks, and 12 weeks after each admission.

**Figure 2:**
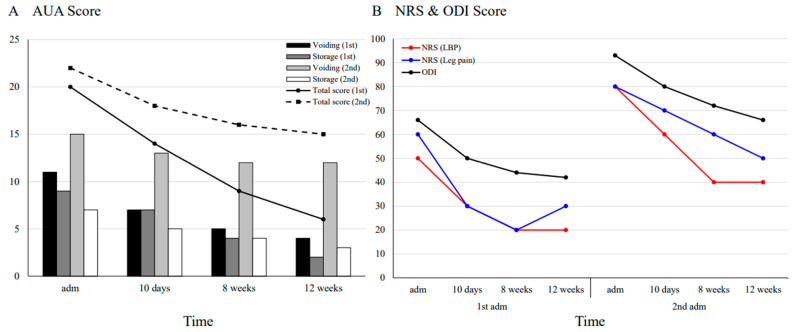
Course of symptoms. (**A**) The prognostic course of lower urinary tract dysfunction (LUTD) severity is assessed using the American Urological Association urinary symptom score, which evaluates symptoms exhibited over the past month. However, at the 10-day assessment, the score reflected symptom changes over the initial 10 days of treatment. (**B**) Changes in scores on the numeric rating scale for low back pain and leg pain, as well as the Oswestry Disability Index for physical function, are evaluated. AUA—American Urological Association; LBP—low back pain; NRS—numeric rating scale; ODI—Oswestry Disability Index.

As shown in Figure 2A, the total AUA urinary symptom score steadily decreased during the first admission, reaching six points at 12 weeks (from 20 [severe] to 6 [mild]). In contrast, the improvement was less pronounced during the second admission (from 22 [severe] to 15 [moderate]). A detailed analysis revealed that during the first admission, both voiding and storage symptoms showed consistent improvement, whereas during the second admission, voiding symptoms decreased by only three points. Discogenic pain and functional disability, evaluated using the NRS and ODI, showed similar trends at both admissions. Pain levels in the lower back and leg decreased by approximately 50% (from an average of 55 to 25 and 80 to 45 during the first and second hospitalizations, respectively), and functional disability scores improved by approximately two-thirds (from 66 to 42 and 93 to 66, respectively) at the 12-week follow-up compared to baseline (Figure 2B). No adverse events were observed during the treatment.

## 3. Discussion

Compression of the cauda equina by a herniated disc can lead to neurological symptoms in the lower limbs and, in severe cases, loss of bowel and bladder control, often necessitating surgical intervention [15]. However, conservative management may be appropriate in mild cases. This report describes a patient with discogenic pain and LUTD who maintained independent ambulation and micturition and was admitted for conservative treatment. The therapeutic effects of PTNS were evaluated by analyzing the outcomes of two separate hospitalizations, each characterized by distinct presentations of HIVD and NB symptoms.

The mechanism underlying PTNS efficacy in CNS-derived LUTD is hypothesized to involve the enhanced regulation of micturition centers in the cortex, pons, and spinal cord via the sciatic nerve [16]. Ascending stimuli from the tibial nerve activate the pontine storage and micturition centers, modulating their rhythm by regulating autonomic nerves, including the pelvic and hypogastric nerves [17]. This case suggests a distinct therapeutic mechanism, as it involves peripheral nerve injury resulting from HIVD. Since bladder functional modulation relies on indirect stimulation of afferent pathways affecting detrusor activity, particularly involving the S2-4 roots, mechanical nerve compression from lumbosacral HIVD and the associated inflammatory response may directly or indirectly impact bladder function [18]. A previous preclinical study demonstrated that PTNS improved bladder storage capacity by delaying voiding onset through an inhibitory effect on bladder afferent signalling at the peripheral level [19]. In addition to its well-known central regulatory effects, this finding suggests that PTNS may provide a therapeutic intervention at the peripheral nerve level for bladder symptoms caused by peripheral nerve compression.

In this case, both storage and voiding functions steadily improved during the first hospitalization, whereas the second hospitalization was marked by a slower recovery of voiding function (Figure 2A). The sacral nerve-derived pelvic splanchnic and pudendal nerves, which regulate the detrusor muscle and external sphincter, respectively, play key roles in voiding function [20]. The difference in recovery patterns between the two hospitalizations may reflect the progression of HIVD because the herniated disc during the second hospitalization exhibited inferior migration and spinal canal stenosis, causing increased sacral nerve compression (Figure 1B). This finding suggests that variations in the location and severity of disc compression may influence voiding dysfunction and potentially alter the efficacy of PTNS, highlighting the need for further research to elucidate the underlying mechanisms.

In addition to HIVD severity, disease duration (acute vs. chronic state) may influence the clinical effectiveness of PTNS. Preclinical and clinical studies of electronic stimulation at SP6 for acute urinary retention have demonstrated bladder pressure-reducing effects [21]. In contrast, evidence supporting PTNS efficacy in chronic urinary retention is limited, with some studies suggesting lower effectiveness [22]. NB associated with disc herniation often begins as acute bladder overactivity that progresses to reduced bladder sensitivity and detrusor atrophy in chronic stages [6]. In the present case, during the second hospitalization for chronic disc herniation, the weakened bladder musculature suggested a slower improvement in voiding function with PTNS therapy. Further research is needed on the relationship between discogenic bladder histopathology, LUTD, and PTNS efficacy in chronic HIVD with NB.

In both hospitalizations, daily PTNS therapy resulted in a significant improvement in the AUA symptom score within 10 days (Figure 2A), a faster response than the 5–7 weeks typically required for CNS-related conditions [10]. Unlike previous studies that applied PTNS weekly, this study involved daily PTNS sessions using acupuncture needles (30-gauge) instead of the standard 34-gauge needles, providing greater stimulation intensity [12]. This method is supported by previous research demonstrating that repetitive peripheral magnetic stimulation in patients with HIVD-induced nerve damage resulted in significant functional improvements [23]. Additionally, unlike conventional acupuncture, tibial nerve stimulation was confirmed by observing large toe flexion, ensuring a reproducible neuromodulatory intervention. This approach may have facilitated earlier clinical improvement, consistent with a previous clinical trial on overactive bladders, which suggested that more frequent PTNS sessions could yield superior outcomes [24].

This study has several limitations. First, objective data such as urodynamic tests and ultrasound-based post-void residual volume measurements were unavailable, as the study relied solely on survey data regarding patients’ LUTD symptoms. This reliance on subjective measurement may introduce bias, such as the Hawthorne effect. Secondly, PTNS therapy was combined with conservative lumbar treatment for pain management, including acupuncture and herbal medicine, during both hospitalizations. In addition, bethanechol and tamsulosin were administered during the second hospitalization, making it difficult to assess the isolated effects of PTNS. Pharmacological and invasive lumbar treatments for HIVD may have also influenced bladder function.

Nevertheless, this case report presents the first evaluation of the effects of PTNS on LUTD caused by HIVD with nerve compression, identified using diffusion tensor imaging, a MRI sequence, strengthened on detecting peripheral nerve pathologies [25]. Although further mechanistic studies and high-quality clinical trials are necessary, our findings suggest that PTNS may serve as a conservative and complementary treatment for bladder dysfunction associated with peripheral nerve injury. Future research should validate these findings through clinical studies assessing the effect of traditional and high-frequency PTNS for NB symptoms caused by HIVD. Additionally, investigations should evaluate the impact of HIVD chronicity, the severity of detrusor atrophy, and the efficacy of PTNS in other peripheral nerve injury-related conditions. This approach may be particularly beneficial for patients who cannot generate intra-abdominal or detrusor pressure, require bed rest, prefer non-invasive treatment, or are unsuitable for catheterization.

## 4. Conclusions

Our key finding is that a 10-day intensive PTNS treatment using 30-gauge needles alleviated NB symptoms caused by HIVD. Additionally, while both storage and voiding functions improved in cases of relatively mild HIVD, voiding function recovery was delayed in severe cases. Large-scale clinical trials are needed to validate these findings, along with further research to refine PTNS application methods and evaluate the relationship between HIVD severity, its characteristics, and treatment efficacy.

## Figures and Tables

**Table 1:** Components of routine conservative treatment.

Items	Treatment Details
Acupuncture	
Acupuncture	Acupuncture and intramuscular Shinbaro 2 * injection therapy are conducted twice daily on ashi-points in the low back
Pharmacopunture	
Herbal medicine	
Chungshinbaro-pill	85 g daily, component (*Poria cocos*, *Panax ginseng*, *Acyranthes bidentata*, *Asini qelatinum*, *Rehmannia glutinosa*, *Cervus elaphus*, *Apis cerana*, *Cibotii rhizoma*, *Eucommia ulmoides*, *Acyranthes bidentata*, *Ledebouriella seseloides*, *Eleutherococcus sessiliflorus*, *Scolopendra subspinipes*, *Atractylodes japonica*, and *Cibotii rhizoma*)
Others	
Thermotherapy	Moxibusion, infra-ray and hot pack treatment are applied to the lower back twice daily

* The solution comprised nine dried wild herbs—Cibotium barometz rhizome, Saposhnikovia divaricata root, Eucommia ulmoides stem bark, Acanthopanax sessiliflorus stem and root, Ostericum koreanum rhizomes and roots, Angelica pubescens root, Achyranthes japonica root, Scolopendra subspinipes, and Paeonia albiflora root—were extracted with 70% ethanol.

## Data Availability

The data presented in this study are available upon reasonable request from the corresponding author. The data are not publicly available owing to privacy and ethical restrictions.
